# Contact lenses as novel tear fluid sampling vehicles for total RNA isolation, precipitation, and amplification

**DOI:** 10.1038/s41598-024-62215-8

**Published:** 2024-05-22

**Authors:** Nikolay Boychev, Seokjoo Lee, Vincent Yeung, Amy E. Ross, Liangju Kuang, Lin Chen, Reza Dana, Joseph B. Ciolino

**Affiliations:** 1 Department of Ophthalmology, Schepens Eye Research Institute, Massachusetts Eye and Ear, and Harvard Medical School, Boston, USA; 2 Department of Optometry and Visual Science, West China Hospital, Sichuan University, Chengdu, Sichuan China; 3 Department of Ophthalmology, Affiliated Hospital of Zunyi Medical University, Zunyi, Guizhou China

**Keywords:** Tear fluid, RNA, Contact lenses, Housekeeping genes, Biomarkers, Ocular diseases, Biochemistry, Biological techniques, Biotechnology, Chemical biology, Genetics, Molecular biology, Biomarkers, Medical research, Chemistry, Materials science

## Abstract

The tear fluid is a readily accessible, potential source for biomarkers of disease and could be used to monitor the ocular response to contact lens (CL) wear or ophthalmic pathologies treated by therapeutic CLs. However, the tear fluid remains largely unexplored as a biomarker source for RNA-based molecular analyses. Using a rabbit model, this study sought to determine whether RNA could be collected from commercial CLs and whether the duration of CL wear would impact RNA recovery. The results were referenced to standardized strips of filtered paper (e.g., Shirmer Strips) placed in the inferior fornix. By performing total RNA isolation, precipitation, and amplification with commercial kits and RT-PCR methods, CLs were found to have no significant differences in RNA concentration and purity compared to Schirmer Strips. The study also identified genes that could be used to normalize RNA levels between tear samples. Of the potential control genes or housekeeping genes, GAPDH was the most stable. This study, which to our knowledge has never been done before, provides a methodology for the detection of RNA and gene expression changes from tear fluid that could be used to monitor or study eye diseases.

## Introduction

Tears serve as the first barrier between the external environment and the eye. The tear fluid is crucial to ocular surface homeostasis by its functional role in microbial defense, wound healing, and maintenance of inflammatory processes^1^. Tears protect and maintain ocular health^2^ by lubricating the ocular surface, providing oxygen and electrolytes to the cornea, increasing the refractive power, and protecting the eye from environmental debris; making it a vital and complex body fluid^3,4^. The secretion and structure of tears are derived from various secretory units (e.g., lacrimal and meibomian glands, conjunctival and corneal cells). Tear secretion is normally a highly regulated and stable process^5,6^ that has been classified as either basal, reflex, or psycho emotional^7,8^. Each type of tear secretion can be altered with the pathogenesis of diseases^7^. Thus, tear fluid also acts as a valuable source for biomarkers indicative of the state of the eye^1,9–17^. For instance, its proximity to the disease site on the ocular surface gives tears a distinct advantage, making it an ideal fluid for evaluating biomarkers for various ocular surface diseases^18–20^.

In the context of tear fluid nucleic acids, RNA has been found to be affected by eye diseases such as wound healing and infections^9,10^. While studies have identified an array of proteins in tears that have long been linked as biomarkers for cancer, neurological disorders, and ocular diseases^11–17^, the tear fluid remains a largely unexplored biomarker source of RNA and relatively few studies have reported isolating ocular RNA from tears^21–26^. Compared to tear protein biomarkers, tear RNA-based biomarkers could be present earlier in the disease process because they reflect changes in gene expression that occur prior to protein expression^27^. The most researched example is in the case of dry eye disease, where changes in the expression levels of several inflammatory-related tear fluid miRNAs were found to be altered compared to healthy controls^28–32^. Therefore, secreted tear fluid may reflect the pathophysiological state of the cell or tissue of origin, and tear RNA-based biomarkers may allow for earlier intervention and management of ocular diseases.

There are several commercially available nucleic acid-based kits that have been cleared or approved by the U.S. Food & Drug Administration’s (FDA) Center for Devices and Radiological Health^33^ for measuring biofluid samples such as blood, serum, plasma, urine, saliva^34–37^, or cerebrospinal fluid^38,39^, but these are not standardized, and none are specific to tear fluid. These kits analyze differences in the sequence, configuration, or expression of RNA to diagnose diseases, identify pathogens, or determine genetic carrier status^33^. Although the RNA biomarker discovery from tear fluid could be promising, the current sample isolation practices in collection, storage, and analyses are challenging^11,18,40–46^. In general, RNA recovery is more difficult with tears compared to other biological fluids or tissue because the tear fluid volume is limited^11,18,40,45,46^. Therefore, there may not be enough tear volume to analyze using newer point-of-care nucleic acid tests such as CRISPR, SELEX, or next-generation sequencing^47^, which are usually used to analyze other biological samples, such as blood or tissue. Moreover, the RNA recovery rate may be sufficient with current methods when amplified by Real-time-PCR (RT-PCR), but the quality may not be acceptable. Thus, protocols for tear fluid RNA extraction, processing, and PCR analysis are prone to data variability, which could make it difficult to compare results across studies.

Contact lenses (CLs) are an FDA-approved medical device worn by more than 140 million people worldwide^44,48^ and a CL-based tear collection method for genetic analyses may offer a relatively non-invasive and patient-friendly approach for obtaining samples. While CLs have been described as a tear fluid collection method for analyzing lipids^49,50^, lysozyme^44,49^, dopamine^51^, and *Staphylococcus aureus*^52^, there has been little research on the use of CLs as a viable option to collect tear fluid in vivo for RNA extraction. Here, we investigated the use of daily CLs for RNA collection from tears. We evaluated how the duration of CL wear affects RNA isolation. RNA collection from CLs was compared to standardized strips of filter paper, e.g., Schirmer Strips, which is one of the most popular tear fluid collection methods of choice^43^. We also performed RT-PCR normalization experiments of GAPDH, ACTB, and HPRT1 housekeeping genes to rank their expression stability. The method described here could be used by researchers to detect RNA and gene expression changes from tears, which could be used to monitor or study eye diseases. We have addressed several factors on using CLs for tear fluid RNA extraction and highlighted challenges that remain with the aim of utilizing tear fluid as a prospective point-of-care for future diagnostic biomarkers in eye care.

## Results

### Evaluation of nucleic acid recovery from CL wear

**Figure 1:**
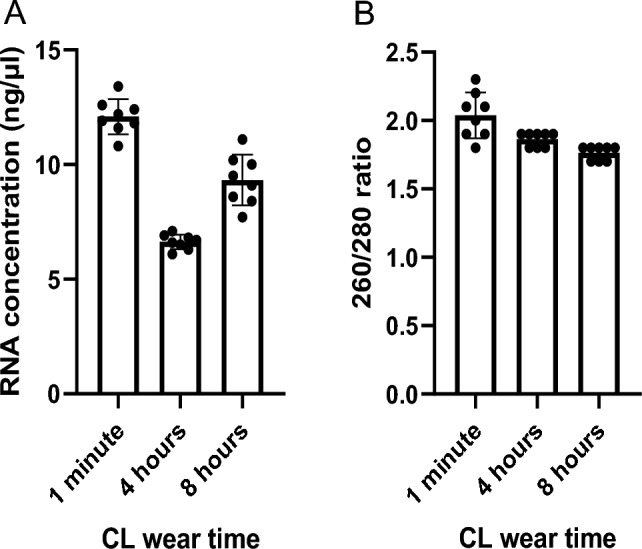
Total RNA concentration and RNA purity analysis for RNA extracted from CL following wear time of 1 min, 4 or 8 h. (**A)** 1-Day Acuvue® Moist CL wear modality (1 min, 4 and 8 h) comparison for total RNA extracted from the tear fluid of healthy rabbits. (**B)** Purity of total RNA from **A** was measured using the ratio of absorbance at 260/280 nm for each individual time point and quantified with NanoDrop 2000. n = 8/time point.

To investigate whether nucleic acids were isolated successfully from rabbit CL samples, we used a modified RNA isolation protocol and adhered to the guidelines for assessment of nucleic acid purity^53,54^. We performed RNA isolation from CLs collected at different timepoints: 1 min, 4 h and 8 h. We found no differences in RNA concentration (ng/µL) from isolated RNA CL samples ranging from 1 min (12 ± 1.6 ng/µl), 4 h (6.7 ± 0.6 ng/µl), or 8 h (9.5 ± 1.7 ng/µl) (P > 0.05) (Fig. 1A). To explore nucleic acid purity, we measured the 260/280 nm (protein/phenol contamination) (Fig. 1B) and 260/230 nm (EDTA/phenol contamination) (Supplemental Fig. 1) ratios of absorbance. The 260/280 nm ratios from 1 min (2 ± 0.2), 4 h (1.9 ± 0.1), or 8 h (1.8 ± 0.1) rabbit CL samples were all accepted to be “pure” for nucleic acid isolation (range of 1.7–2.1)^55^. There were minimal differences for the 260/230 nm ratios for the time points tested and they were all below the expected range. For example, at 1 min the 260/230 nm ratio was 0.5 ± 0.08 and the ratio range accepted to be “pure” for nucleic acid isolation is 2.0–2.2^55^. Collectively, the data suggests that we have developed a nucleic acid extraction methodology from CL samples, with minimal difference in RNA concentration in duration of CL wear.

### Comparing nucleic acid recovery from CLs and Schirmer Strips

**Figure 2:**
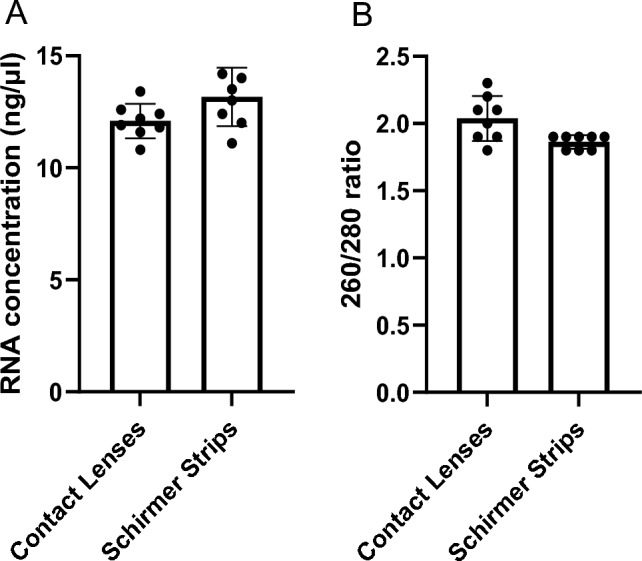
Comparison of total RNA concentration and purity for daily CL wear versus Schirmer Strips following 1 min collection**.** (**A**) 1-Day Acuvue® Moist CL wear and Schirmer Strips comparison for total RNA extracted from the tear fluid of healthy rabbits. (**B**) Purity of total RNA from **A** was measured using the ratio of absorbance at 260/280 nm and quantified with NanoDrop 2000. n = 8/group.

To assess the effectiveness of nucleic acid recovery from worn CLs, comparisons were made to conventional Schirmer Strips that were worn separately for 1 min and on different days. Fresh tear fluid and nucleic acid were isolated from rabbit CLs and Schirmer Strips using our modified nucleic acid protocol at the 1 min timepoint. We show no differences in RNA concentration from isolated RNA CL (12 ± 1.6 ng/µl) and Schirmer Strip (13.5 ± 1.8 ng/µl) samples (p  > 0.05) (Fig. 2A). The 260/280 nm ratio difference between CLs (2.0 ± 0.2) and Schirmer Strips (1.9 ± 0.1) was negligible, with both indicative of a “pure” nucleic acid sample (Fig. 2B). Meanwhile, all observed 260/230 nm ratios were lower than the expected ‘pure’ range with minimal differences between the CLs (0.7 ± 0.09) and Schirmer Strips (0.5 ± 0.08) (Supplemental Fig. 2). The data show similarities in the quantity and purity of isolated nucleic acid between CL and Schirmer Strip samples.

### Identifying the optimized storage conditions for tear fluid cells from CLs

**Figure 3:**
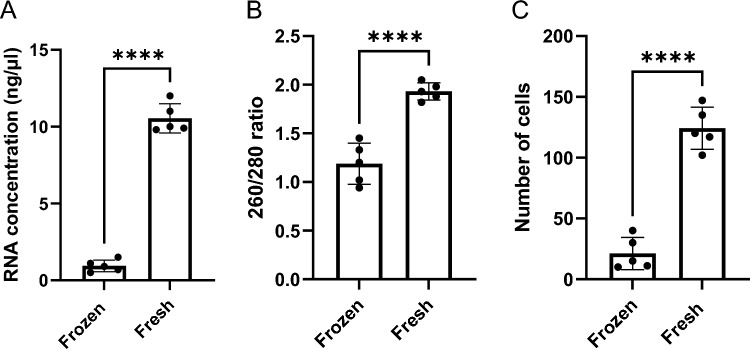
Importance of using fresh cells for RNA yield and quality**.** (**A**) RNA concentrations (ng/μL) were measured using a NanoDrop 2000 Spectrophotometer for fresh and frozen cells from the tear fluid of healthy rabbits. (**B**) 260/280 nm absorbance ratio. (**C**) Cell numbers were measured using an automated cell counter before and after freezing. n = 5/group, where ****(p < 0.0001) represented a significant difference using an unpaired t-test.

It has been reported that storage conditions of tear fluid samples can influence the presence of biomolecules (proteins, lipids, or nucleic acids)^46^. Since the differences in nucleic acid concentration between Schirmer Strips and CLs were negligible, we next assessed whether storage conditions would impact the quality of isolated nucleic acid from fresh or frozen CL samples. Isolated nucleic acid samples from fresh CL samples showed higher RNA concentration compared to frozen samples (10.5 ± 0.8 ng/µl vs. 0.9 ± 0.3 ng/µl, *p* < 0.0001) (Fig. 3A). Similarly, we observed that fresh CL RNA samples showed a higher “purity” in the 260/280 nm ratio compared to frozen CL RNA samples (1.9 ± 0.1 vs. 1.2 ± 0.2,* p* < 0.0001) (Fig. 3B). The 260/230 nm ratios for the fresh RNA CL samples were also significantly higher than the frozen RNA CL samples (0.6 ± 0.1 vs. 0.2 ± 0.04,* p* < 0.0001) (Supplemental Fig. 3). Interestingly, we see that the total number of isolated cells from fresh CL samples was significantly lower compared to frozen CL samples (124.9 ± 13.6 vs. 23.9 ± 10.9, *p* < 0.0001) (Fig. 3C). This data suggests that isolated RNA from CL tear samples that are stored fresh would increase the yield and purity, compared to frozen CL tear samples.

### Effects of fresh vs. frozen RNA CL tear samples on housekeeping gene expression

**Figure 4:**
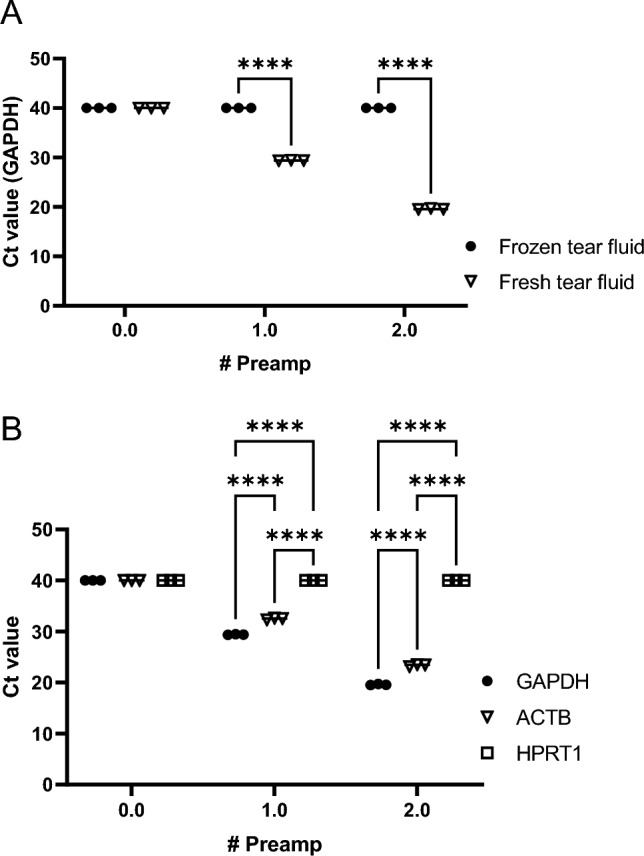
(**A**) Optimized normalization of GAPDH. Comparison of GAPDH Ct values from fresh and frozen RNA cells in the tear fluid of healthy rabbits, after RT-PCR. Results were obtained from non-amplified (0.0), one-time preamplified (1.0), and two-times preamplified (2.0) cDNA. n = 3/group, where ****(p < 0.0001) represented a significant difference using two-way ANOVA. (**B**) Representative precipitation and amplification assessment of housekeeping genes for RT-PCR**.** Comparison of Ct values for GAPDH, ACTB, and HPRT1 from fresh RNA tear fluid cells of healthy rabbits, after RT-PCR. Results were obtained from non-amplified (0.0), one-time preamplified (1.0), and two-times preamplified (2.0) cDNA. n = 3/group, where ****(p < 0.0001) represented a significant difference using two-way ANOVA.

Considering the differences in RNA yield and purity of fresh vs. frozen CL tear samples, we next determined the expression of GAPDH, an extensively used housekeeping gene recognized for its broad coverage in diverse species and cell types. As RNA samples were limited, we performed an RNA preamplification step prior to qPCR to enhance their sensitivity and subsequent analysis. We observe that RNA extracted from frozen CL tear samples did not yield quantifiable cycle threshold (Ct) values with the cutoff at Ct 40 (Fig. 4A), despite two rounds of preamplification. Whereas we observe a significant decrease in Ct value with one round of preamplification from fresh CL RNA samples compared to frozen CL RNA samples (29.42 ± 0.05 vs. 40 ± 0.00,* p* < 0.0001). These findings were similarly replicated with two rounds of preamplification with fresh CL RNA samples (19.60 ± 0.08, *p* < 0.0001) compared to frozen CL RNA samples.

We next explored the expression profiles of other housekeeping genes that included ACTB and HPRT1 in reference to GAPDH. Compared with GAPDH that had the lowest Ct values following one or two rounds of preamplification, ACTB showed slightly elevated Ct values following one (32.51 ± 0.15, *p* < 0.0001) or two (23.38 ± 0.16, *p* < 0.0001) amplification (Fig. 4B). Yet, the Ct values for HPRT1 did not yield quantifiable cycle threshold Ct values with the cutoff value at Ct 40. Collectively, this data suggests that GAPDH is the suitable housekeeping gene following RNA extraction from fresh CL samples.

### Pathway analysis of GAPDH from fresh RNA CL tear samples

**Figure 5:**
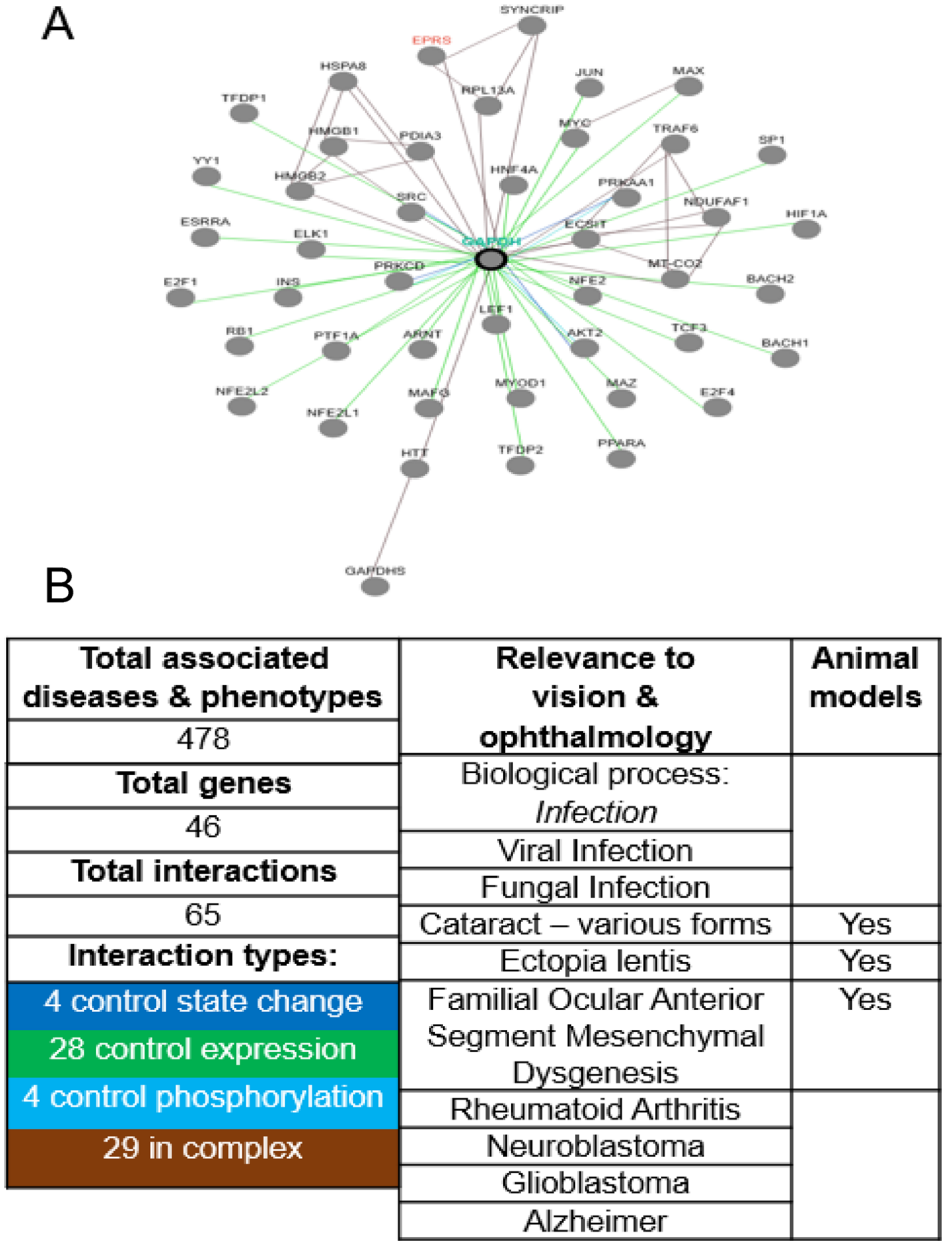
Pathway analysis of GAPDH. (**A**) Interaction pathways. (**B**) Genes, interactions, and associated diseases and phenotypes with animal models specific to eye and vision research**.**

To understand functional insight into the prospective gene pathways from fresh RNA CL tear samples, we show changes in genes from GAPDH expression **(**Fig. 5A**)**. From the shortlisted set of genes, we used the Pathway Commons Network Visualizer (PCViz) and Open Targets Platform to understand their relevance in eye diseases **(**Fig. 5B**)**. GAPDH has the leading expression stability and correlations to biological processes of infection, as well as anterior segment (with existing animal models), systemic, and neurological ocular diseases through the PCViz & Open Targets Platform. Collectively, downstream GAPDH gene analysis suggests they interact with 46 genes (with 65 complex interactions), and broadly with 478 associated diseases and phenotypes. Yet, with an emphasis on anterior segment ocular conditions, there is relevance on cataracts (various forms), ectopia lentis, and familial mesenchymal dysgenesis. Interestingly, ACTB shows some correlation with some biological processes with 616 genes (Supplemental Fig. 4A), whilst HPRT1 had limited correlations with biological processes despite the low Ct values (Supplemental Fig. 4B).

## Discussion

We developed a method (Table 1) to collect RNA from contact lenses (CLs) given the growing interest in using tear biomarkers as a means of diagnosing, treating, and understanding the underlying pathology of ocular diseases. Here, RNA recovery from CLs was indistinguishable from that of Schirmer Strips when tested in healthy rabbit eyes. When we examined whether the duration of CL wear influenced RNA recovery, we found that RNA collection was consistent irrespective of whether lenses were worn for 1 min, 4 or 8 h. In our study, the method of sample storage was found to influence the quality of RNA recovery; and fresh samples yielded superior RNA concentration than frozen samples. We also evaluated potential reference genes that can be used to standardize RNA biomarker collection from CLs and identified GAPDH as the optimal housekeeping gene. To our knowledge, this is the first study to demonstrate that RNA can successfully be extracted from CLs and this method could potentially be used to improve the diagnosis and treatments of ophthalmic diseases.

**Table 1:** Summary of study questions, hypotheses, and outcome measures.

Question	Hypothesis	Outcome measures
Can CLs be used as an ocular RNA biomarker tear sampling vehicle?	CLs are a viable tear fluid collection method for the discovery of RNA biomarkers	Total RNA concentration and purity from standard, commercially available CLs and Schirmer Strips
What is the impact of daily CL wear time on RNA samples collected from tear fluid?	Different modalities of daily CL wear do not affect tear fluid RNA sampling	The potential daily CL wear variations of total RNA concentration and purity at three time points: 1 min, 4 and 8 h
Is the extraction of RNA from tear fluid samples collected using CLs a viable method for gene analyses?	The implementation of a conventional RNA extraction procedure, coupled with effective genomic DNA clearance, can yield adequate amounts of RNA suitable for subsequent cDNA synthesis	RNA concentration, number of RNA cells, ratios of absorbance at 260/230 nm and 260/280 nm to measure quality and purity
What techniques can be applied to detect extremely low concentrations of RNA from tear fluid samples collected using CLs?	A reliable detection of target genes can be achieved by preamplifying cDNA synthesized from low levels of tear fluid RNA collected with CLs using appropriate reagents	RT-PCR was used to measure Ct values of the target genes
Which is the recommended housekeeping gene for gene biomarker analyses from tear fluid in rabbit models?	GAPDH stands as the most ideal housekeeping gene in this context and test model	GAPDH, ACTB, and HPRT1

The path to creating the first guidelines for using CLs in the RNA isolation, precipitation, and amplification from tear fluid samples of healthy rabbits.

Several unexpected findings emerged. Despite different durations of CL wear, minimal differences in RNA concentration were observed, suggesting the potential degradation of RNA molecules is stable for longer than expected. This could be notable for other related RNA molecules (e.g., mRNA, miRNA, tRNA, rRNA) extracted from tear fluid that could be more sensitive^56–58^. RNA extracted from CL samples showed no significant difference in concentration and purity compared to RNA from Schirmer Strips, indicating the feasibility of using CLs for tear fluid collection and RNA extraction from CL wearers. We also found that frozen CL samples yielded non-quantifiable RNA even after two rounds of preamplification, suggesting degradation during the freezing and thawing processes that we employed. While not necessarily surprising, we found that housekeeping genes that underwent two preamplifications obtained enhanced sensitivity to extracted RNA from CL samples by yielding elevated cycle threshold (Ct) values. Non-quantifiable Ct values can be primarily attributed to factors like ice crystal formation and enzymatic activity^59^, which can negatively affect downstream applications relying on high-quality RNA, such as RT-PCR.

The use of housekeeping genes, as a normalization factor in gene expression studies is considered the most accurate method for correcting potential biases in sample collection, starting material, reverse transcriptase, RT-PCR efficiency, and nucleic acid preparation quality^60–62^. However, no single gene can serve as a universal endogenous control for all experimental conditions^60–62^. Of the housekeeping genes we investigated using our RNA isolation method with RT-PCR, GAPDH yielded the highest Ct value, while ACTB had a moderately acceptable Ct value, and HPRT1 did not meet the detection threshold. GAPDH’s gene network may have a greater potential for specificity than ACTB and HPRT1 towards the discovery of novel interaction changes and treatment targets for ocular disorders. Gene interaction refers to the combined role of several genes in determining phenotypic variability that affects biological processes^60–64^. This could be notable for infections and other anterior segment diseases, as we have reported through our use of the PCViz and Open Targets Platform.

The development of a methodology for extracting RNA from tears collected using CLs offers a promising avenue for research and clinical applications in ocular health. Collecting RNA from CLs worn by individuals with existing conditions, especially anterior eye diseases such as dry eye, allergic conjunctivitis, or keratoconus, as well as systemic or neurological disorders, could provide valuable insights into disease mechanisms and biomarker identification^1,9–14,24,28,32,38,39,65^. For instance, RNA extracted from CL tear samples could elucidate gene expression patterns associated with inflammation, infection, or oxidative stress in the ocular surface epithelium.

Conventionally, tear fluid has been collected using various methods^41,43^, of which Schirmer Strips are one of the most preferred options^43^ because the paper strips can collect tear samples within several minutes^66^. Here, we collected Schirmer Strips after 1 min based on past findings indicating that longer sampling durations underestimated actual tear analyte concentrations^66^. Future studies may want to incorporate comparisons with other tear fluid isolation methodologies such as capillary tubes or micropapillary pipettes^43,66^, as well as longer Schirmer Strip sampling of 5 min^66^, for obtaining a more comprehensive evaluation of tear function and ocular surface health.

This study has several limitations and presents other opportunities for future research. There were some variables involved in RNA precipitation that we did not study such as incubation time and temperature; centrifugation force and time; volume ratio of ethanol to RNA solution; and the use of types of cations and coprecipitators^67^. Future studies could investigate whether changing these variables affects RNA recovery or compare this method to the TRIzol protocol^68^. Another area of future research includes comparison of different CL materials, as they may differ in the absorption of tear fluid and the subsequent maintenance of RNA integrity. Because this study was performed in healthy rabbit eyes, further studies are needed to confirm whether these findings, such as the use of GAPDH as the housekeeping gene, remain consistent when studying animal models of disease or human tears.

**Figure 6:**
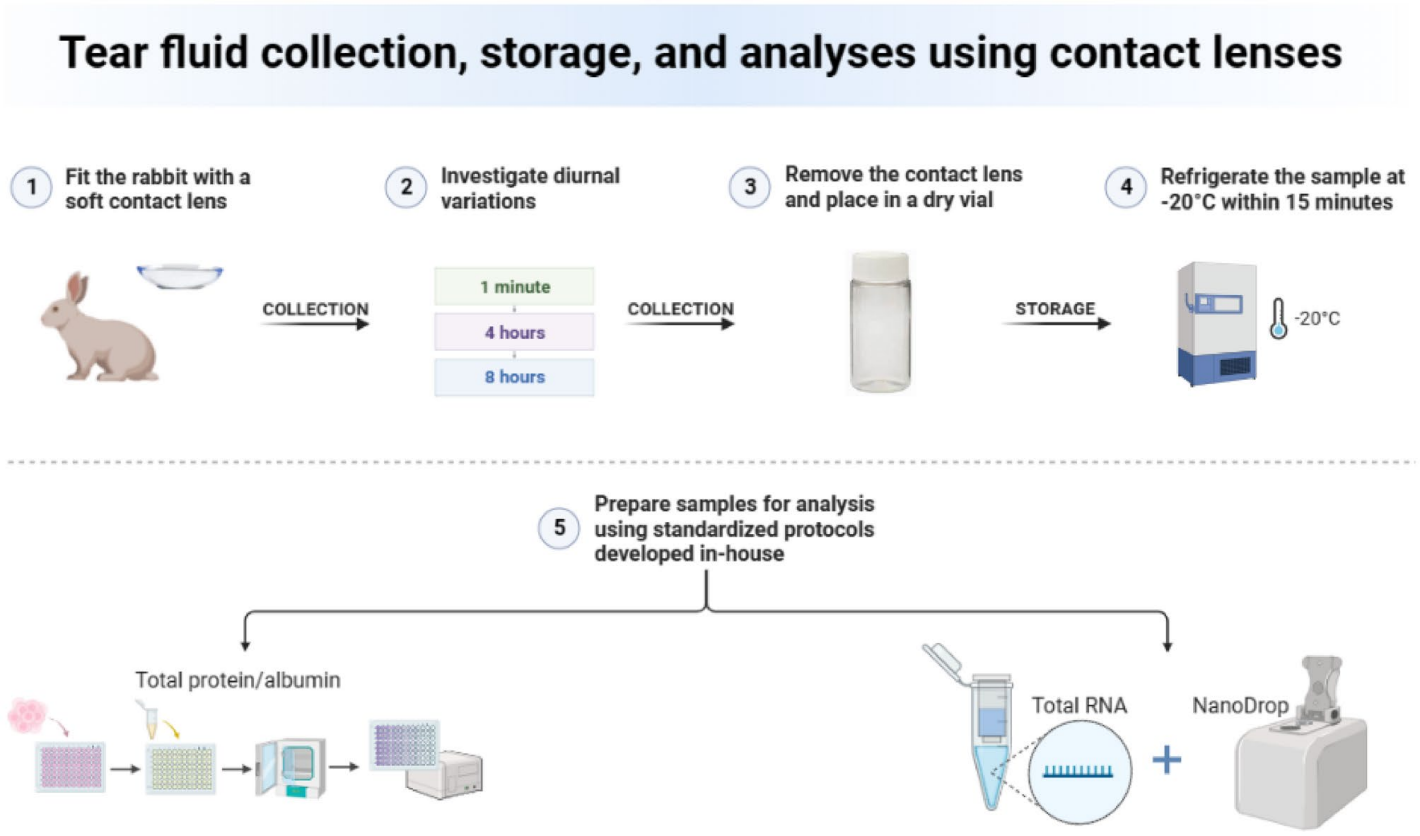
Tear fluid collection, storage, and analyses using contact lenses.

We successfully extracted RNA from contact lenses (CLs), presenting a promising method in enabling reliable detection of target genes. We found that using fresh tear samples and two preamplifications resulted in superior concentration of RNA; these methods may be important for ensuring reliable results, particularly when performing gene expression analysis on tear fluid that is already collected in relatively small volumes (Fig. 6). Moreover, RNA recovery from CLs matched that of Schirmer Strips, regardless of CL wear time. GAPDH emerged as the optimal housekeeping gene for normalization. Further research is needed to validate and refine these findings for wider tear fluid biomarker analysis of ocular diseases.

## Methods

### Animals

A combination of healthy adult male and female New Zealand White rabbits (Charles River Laboratories, Boston MA) weighing an average of 4 kg were used in each group. Their application in animal research and translational scope has been widely documented^69–72^. The rabbits were housed in standard cages in a light-controlled room at a temperature of 23 ± 2 °C, relative humidity of 60 ± 10%, and a 12 h light–dark cycle (6 AM to 6 PM). The rabbits were given food and water ad libitum. The rabbits used for experiments were not sacrificed after use in this study, and therefore, no chemical agent was used for euthanization.

### CLs

The FDA-approved and daily commercial CLs made of etafilcon A hydrogel material (Plano-1-Day Acuvue® Moist, Johnson&Johnson) were chosen, according to previous literature on the different protein capture properties of varying CL materials^44,73,74^.

### Study design

We chose wear times of 1 min, 4 and 8 h (n  = 8/time point) to understand the potential impact of daily CL wear time on the extracted RNA collected from healthy rabbit tears. We also made comparisons with reflex tears collected by Schirmer Strips (TearFlo, HUB Pharmaceuticals) at the 1 min time point (n  =  8) based on past findings^75^. The rabbits were fitted with the CLs without anesthesia in a randomized order and with a minimum of 24 h washout period in between. CLs were fitted and removed at matched times in the day to account for possible wear variations. The collected tears by Schirmer Strips without anesthesia were taken from the contralateral eye that was without a CL.

The Schirmer Strip application followed a previously published methodology^67^. Briefly, a Schirmer Strip was inserted either in the rabbit’s lower conjunctival sac or lateral canthus, until achieving a reading of ≥ 5 mm wetting based on a past recommendation^76^. The Schirmer Strip collection was performed apart of CL wear to ensure there was no confounding effect.

On removal, the CLs were placed in dry vials and Schirmer Strips in dry Eppendorf tubes, without processing since dry storage has been shown to be of importance to tear fluid sampling preservation^51^. The samples were then either directly streamed for total RNA isolation, precipitation, and amplification, or stored at − 20 °C within 15 min. The corneal integrity of each rabbit was verified by an eye care practitioner using a fluorescein staining solution under cobalt blue light.

### Sample preparation and analyses: total RNA isolation, precipitation, and amplification

#### Cell counting

We counted the number of tear fluid cells using an automated cell counter before and after freezing and compared the results. The samples were prepared according to standard protocols and loaded onto the EVE chamber slides. The EVE™ Automated Cell Counter (NanoEnTek, Republic of Korea) was then used to capture images of the cells, and the EVE software was used to analyze the images and generate cell counts. The accuracy of the cell counts was validated by comparison to manual cell counting. 10 μl was utilized with Trypan Blue staining for the manual counting of cells on a hematocytometer (Sigma-Aldrich).

#### RNA extraction

RNA was isolated using a Qiagen RNeasy Mini Kit (Qiagen) according to the manufacturer’s instructions. Briefly, CLs and Schirmer Strips were transferred into new Eppendorf tubes and 1 ml of phosphate-buffered saline (PBS) solution was added to each sample, then left to incubate overnight for minimum of 8 h on a shaker/rocker at 4 °C. The next day, the samples were centrifuged at 4 °C/10,000 RPM for 10 min and the cell pellet was added to the RNA lysis buffer provided with the kit. At this point, the samples allocated for the frozen sample analyses were stored at − 80 °C, while the samples allocated to the fresh sample analyses were processed with the next step. Lysate was transferred to the spin column to allow for RNA binding. RNA was eluted from the column with RNAse-free water or buffer provided with the kit. Sample RNA was treated with DNase to remove any contaminating genomic DNA. The frozen samples stored at − 80 °C were thawed on ice and subsequently centrifuged at 4 °C /10,000 RPM for 10 min to extract the RNA, following the same procedure as fresh samples.

#### RNA quantification and quality control

RNA was quantified with the NanoDrop 2000 Spectrophotometer (ThermoFisher) for concentration and purity. The absorbance ratios of 260/280 nm and 260/230 nm were used to test for purity and contamination. Pure RNA is in the range of 1.7–2.1 for the 260/280 nm absorbance ratio and 2.0–2.2 for 260/230 nm^55^.

#### cDNA synthesis

The samples were either directly streamed for cDNA synthesis or the synthesized cDNA and extracted RNA were stored at − 80 °C. The reverse transcription reaction was performed according to the manufacturer’s instructions. Briefly, RNA with adjusted concentration was incubated with the reagent mixture at 42 °C for 30 min. The reverse transcriptase enzyme was inactivated by incubating the reaction mixture at 95 °C for 3 min. Then, the reaction mixture was chilled on ice and stored at − 20 °C, until further use. An RNase-free tube consisted of the Quantiscript Reverse Transcriptase (1 μl), buffer (1 μl), and Reverse Transcriptase Primer Mix (4 μl). An RNA sample (10 ng/μl) and RNase-free water were combined to a final volume of 14 μl.

#### cDNA preamplification

The cDNA preamplification was performed according to the manufacturer’s instructions (SsoAdvanced PreAmp Supermix, Bio-Rad). We prepared a master mix of the preamplification reaction by multiplying the volume of each component by the number of reactions, considering any excess volume required for pipetting. An RNase-free tube consisted of the SsoAdvanced PreAmp Supermix (25 μl), TaqMan primers (12.5 μl), and cDNA sample (12.5 μl). The cycling conditions for preamplification included an initial denaturation at 95 °C for 3 min to activate the Taq polymerase enzyme, 12 amplification cycles of the denaturation step at 95 °C for 15 s and annealing and extension steps at 58 °C for 4 min. Samples were held at 4 °C until further use. The quality and quantity of the preamplified cDNA were checked using PCR with TaqMan gene-specific primers.

TaqMan primer design and validation are critical steps in the preamplification process. After using the TaqMan Endogenous Control Assay selector tool to identify the most popular endogenous control candidates in the rabbit species and select the most appropriate reference genes for our quantitative PCR experiments, we discovered only five potential targets (Supplemental Table 1).

At least two to three genes are required to validate and test candidate endogenous controls to ensure their suitability for the specific experiments being conducted. Based on previous gene expression reports and potential for human clinical translation^26,60–62,77,78^, we chose the following three TaqMan (ThermoFisher) preamplification primers for rabbit species in the present study: glyceraldehyde 3-phosphate dehydrogenase (GAPDH, Oc03823402_g1), actin beta (ACTB, Oc03824857_g1), and hypoxanthine phosphoribosyltransferase 1 (HPRT1, Oc03399461_m1). We then used the PCViz and Open Targets Platform to analyze the translational scope of these genes in ocular tear fluid research.

#### TaqMan RT-PCR

We prepared the PCR reaction mix by combining the TaqMan Universal PCR Master Mix (Applied Biosystems) of 12.5 μl, 20× TaqMan Primers (GAPDH, ACTB, and HPRT1) of 1.25 μl, cDNA template of 2 μl, and PCR-grade water (Ambion) balanced to 25 μl. The cDNA samples were added to the reaction mix for a final volume of 25 µl. The reaction mix was loaded into the wells of an RT-PCR plate according to the manufacturer’s instructions. The following is our reaction mix protocol. The RT-PCR cycling conditions included an initial denaturation at 95 °C for 30 s, 40 amplification cycles of the denaturation step at 95 °C for 15 s and annealing and extension steps at 60 °C for 1 min. For the negative control, DEPC-treated water was included instead of cDNA to ensure the absence of non-specific amplification or contamination, with the expectation of no detectable amplification signal. For the positive control, cDNA derived from lymphocytes were used, resulting in a Ct value within the expected range, confirming the functionality of the PCR reaction and detection system.

### Statistics

Statistical analyses, including an unpaired t-test, two-way ANOVA, and Bonferroni post hoc tests, were conducted using Prism Software from Microsoft Corp. The values are reported as means ± SD; p < 0.05* indicated statistical significance; statistical significance between groups was denoted ****(p < 0.0001). There is no validated evidence to warrant the reporting of sex- and gender-based analyses.

The RT-PCR data was analyzed using the Eppendorf Realplex2 96-Well Mastercycler epGradient S PCR ThermoCycler software (Eppendorf) by calculating the relative expression of the target gene normalized to a housekeeping gene or a reference gene.

### Ethics

This study was conducted at the Schepens Eye Research Institute of Massachusetts Eye and Ear and Harvard Medical School Department of Ophthalmology. Institutional Animal Care and Utilization Committee ethics and protocol (#2020N000193) approvals were obtained. All animals were treated according to the US Public Health Service Policy on Humane Care and Use of Laboratory Animals, Association for Research in Vision and Ophthalmology Statement for the Use of Animals in Ophthalmic and Vision Research (ARVO Handbook, 1993), and in compliance with the ARRIVE guidelines. Thus, we can confirm that all experiments were performed in accordance with relevant guidelines and regulations.

### Supplementary Information

Supplementary Information.

## Data Availability

The datasets used and analyzed during the current study are available from the corresponding author on reasonable request.

## Abbreviations

FDA: U.S. Food and drug administration’s
RT-PCR: Real-time-PCR
CLs: Contact lenses
Ct: Cycle threshold
PCViz: Pathway commons network visualizer
PBS: Phosphate-buffered saline
