# Supplementary material for: Contact lenses as novel tear fluid sampling vehicles for total RNA isolation, precipitation, and amplification
Source: Sci Rep. 2024 May 22;14:11727. doi: 10.1038/s41598-024-62215-8 (PMC11111455; doi:10.1038/s41598-024-62215-8)
Supplement: Supplementary file 1 — Supplementary Information. [file 41598_2024_62215_MOESM1_ESM.docx]

**Supplemental Figure 1 Total RNA concentration and RNA purity analysis for RNA extracted from CL following wear time of 1 minute, 4 hours, or 8 hours.** Purity of total RNA from the main Figure 1A was measured using the ratio of absorbance at 260/230nm for each individual time point and quantified with NanoDrop 2000. n=8/time point.

**Supplemental Figure 2 Comparison of total RNA concentration and purity for daily CL wear versus Schirmer Strips following 1 minute collection.** Purity of total RNA from the main Figure 2A was measured using the ratio of absorbance at 260/230nm and quantified with NanoDrop 2000. n=8/group.

**Supplemental Figure 3 Importance of using fresh cells for RNA yield and quality.**

Purity of frozen and fresh RNA cells from the main Figure 3A was measured using the ratio of absorbance at 260/230nm and quantified with NanoDrop 2000. n=5/group.


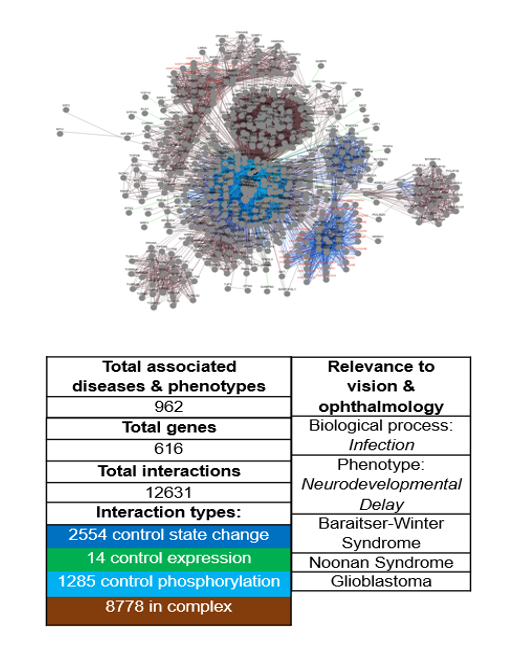

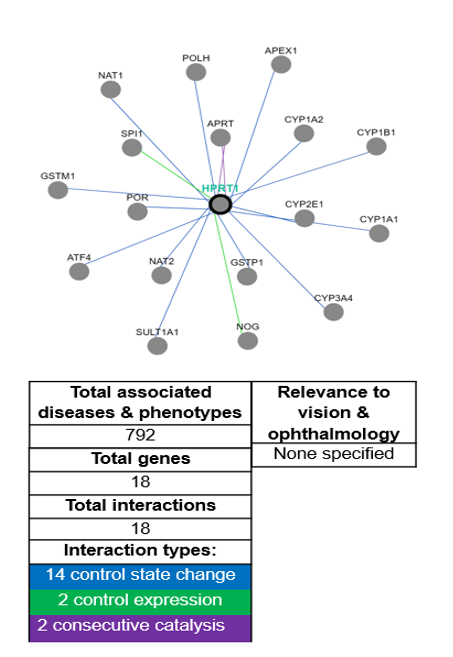


A

B

**Supplemental Figure 4 Housekeeping gene comparisons of the involved interaction pathways, diseases, and phenotypes specific to eye and vision research.** *Supplemental Figure 4A.* ACTB. *Supplemental Figure 4B.* HPRT1.

| **Gene Symbol** | **Aliases** | **Chromosome Location** | **ID’s** |
| --- | --- | --- | --- |
| GAPDH | G3PD, GAPD, HEL-S-162eP | 12p13.31 | UniProt: [P04406](http://www.uniprot.org/uniprot/P04406)  Gene: [2597](http://www.ncbi.nlm.nih.gov/gene?term=2597) |
| ACTB | BRWS1, PS1TP5BP1 | 7p22.1 | UniProt: P60709  Gene: 60 |
| HPRT1 | HPRT, HGPRT | Xq26.2-q26.3 | UniProt: P00492  Gene: 3251 |
| CDKN1B | KIP1, MEN4, CDKN4, MEN1B, P27KIP1 | 12p13.11 | UniProt: P46527  Gene: 1027 |
| PPIA | CYPA, CYPH, HEL-S-69p | 7p13 | UniProt: P62937  Gene: 5478 |

**Supplemental Table 1 Summary of the five potential normalization genes in rabbit species that can be used to establish endogenous controls for gene expression research.**
